# Primary Peritoneal Carcinosarcoma: A Case Report

**DOI:** 10.3389/fsurg.2021.707929

**Published:** 2021-08-19

**Authors:** Logan Erz, Brandon Smith, Brandon Larson, Truong Ma

**Affiliations:** Summa Akron City Hospital, Akron, OH, United States

**Keywords:** peritoneal carcinosarcoma, peritoneal carcinoma, Mullerian tumor, multiple Mullerian tumor, carcinosarcoma

## Abstract

**Introduction:** Carcinosarcoma, also known as malignant mixed Mullerian tumor (MMMT) is a malignant biphasic neoplasm consisting of carcinomatous and malignant non-epithelial components of mesenchymal origin. MMMTs typically arise from the female genital tract in patients over 40 years old. Primary extragenital MMMTs are extremely rare with published literature totaling 40 reported cases. The primary peritoneal carcinosarcoma is an aggressive tumor as patients with this tumor have an average survival of 7.6 months. Surgical debulking is the mainstay of treatment for these tumors and systemic chemotherapy is advised in all cases.

**Case:** A 48-year-old Amish female presented with 5 day history of bloating and abdominal pain superimposed on a 1 year history of worsening fatigue and intermittent bloody bowel movements. She was found to have a pelvic mass on physical exam. Computed tomography scan of the abdomen and pelvis that demonstrated stricturing of the sigmoid colon, and a large multi-cystic mass in the midline pelvis measuring 12.5 × 9.9 × 11.7 cm. Colonoscopy showed stenosis due to external compression without intraluminal lesion. CEA and CA 125 levels were elevated and CA 19-9 was normal. Exploratory laparotomy was performed with en-bloc resection of a 15 cm mass originating from the sigmoid colon mesentery with several other small tumor deposits throughout the mesentery. Pathology diagnosed primary peritoneal carcinosarcoma Mullerian-type with three positive lymph nodes.

**Conclusion:** Malignant mixed Mullerian tumor (carcinosarcoma) caries a universally grim prognosis. Herein, we report a unique case of primary peritoneal carcinosarcoma and discuss the work-up and surgical management of this rare tumor.

## Introduction

Carcinosarcoma is a malignant biphasic neoplasm consisting of carcinomatous and malignant non-epithelial components of mesenchymal origin, is also known as malignant mixed Mullerian tumor (MMMT). Usually, MMMTs arise from the female genital tract, including the ovaries, uterus, and fallopian tubes. Those that arise from extragenital locations, such as the peritoneum, serosal surface of the colon, and omentum, are extremely rare (1). There are ~40 cases reported by literature review (2). The primary peritoneal carcinosarcoma is an aggressive tumor. Patients with tumor arising from colonic/rectal peritoneum had an average survival of 7.6 months (3). Complete cytoreduction surgery is the mainstay of treatment for primary peritoneal carcinomas. Systemic chemotherapy is advised in all cases because of the early spreading of these tumors. The majority of cases are described in women over the age of 40 (4).

## Case Presentation

**Figure 1:**
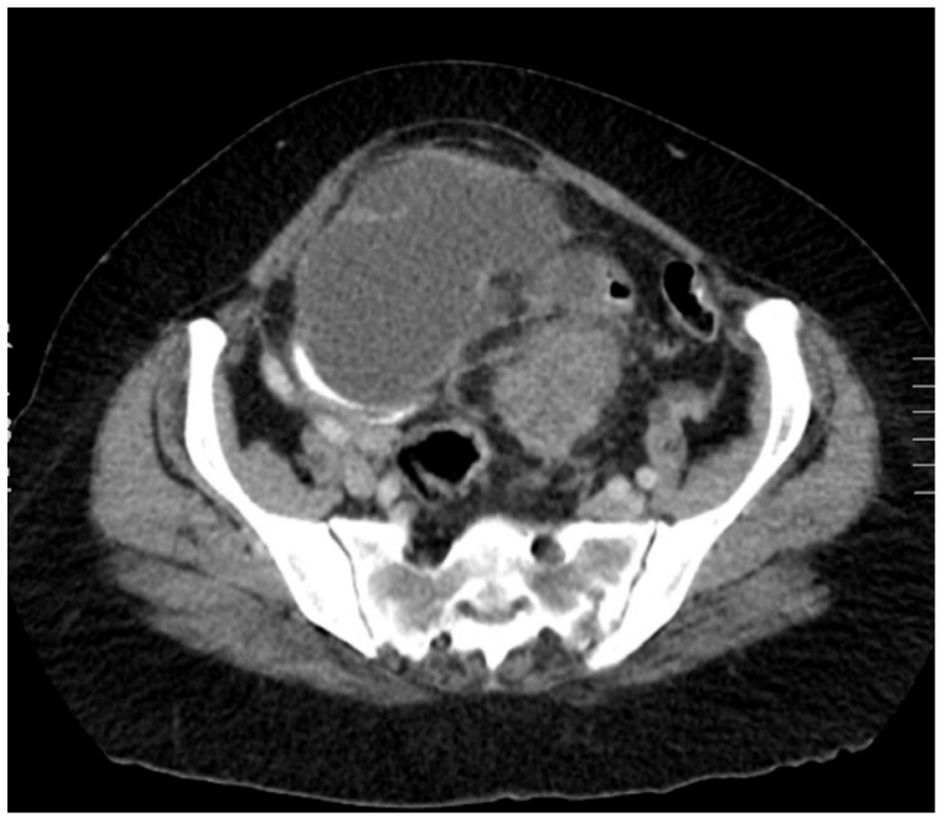
Axial CT with mass measuring ~11 cm × 10 cm.

**Figure 2:**
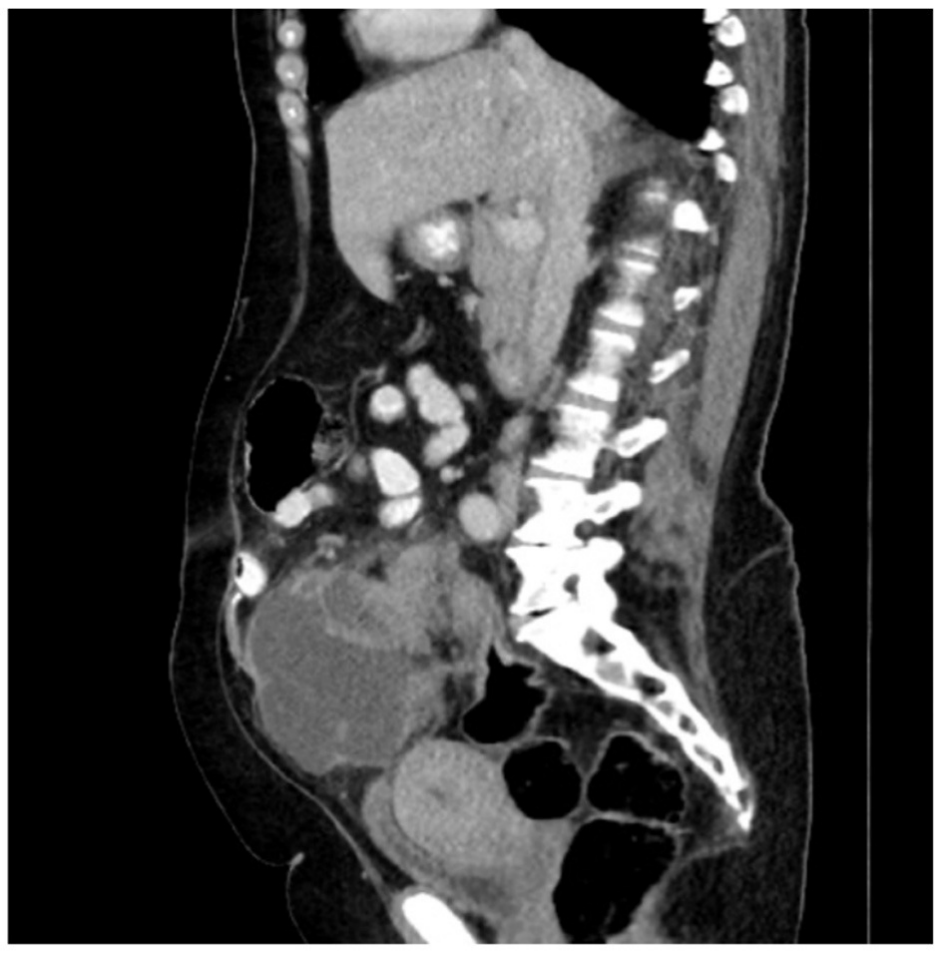
Sagittal CT with mass measuring ~9 cm × 7.5 cm.

**Figure 3:**
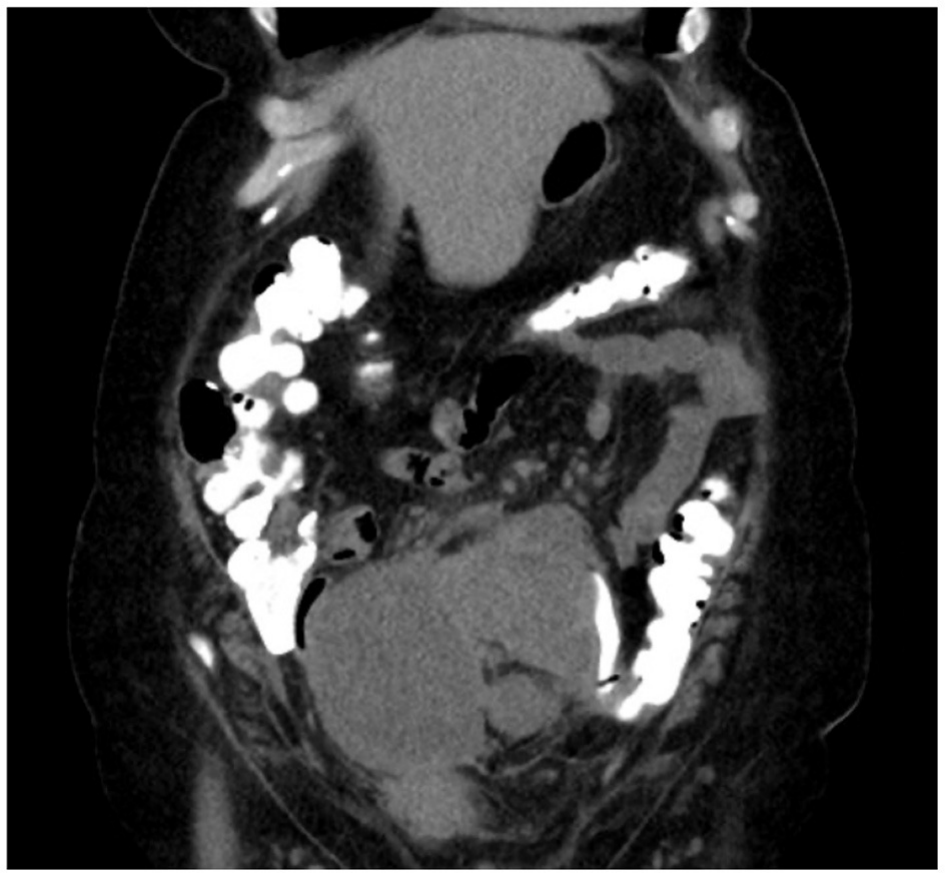
Coronal CT with mass measuring ~12.5 cm × 8.5 cm.

**Figure 4:**
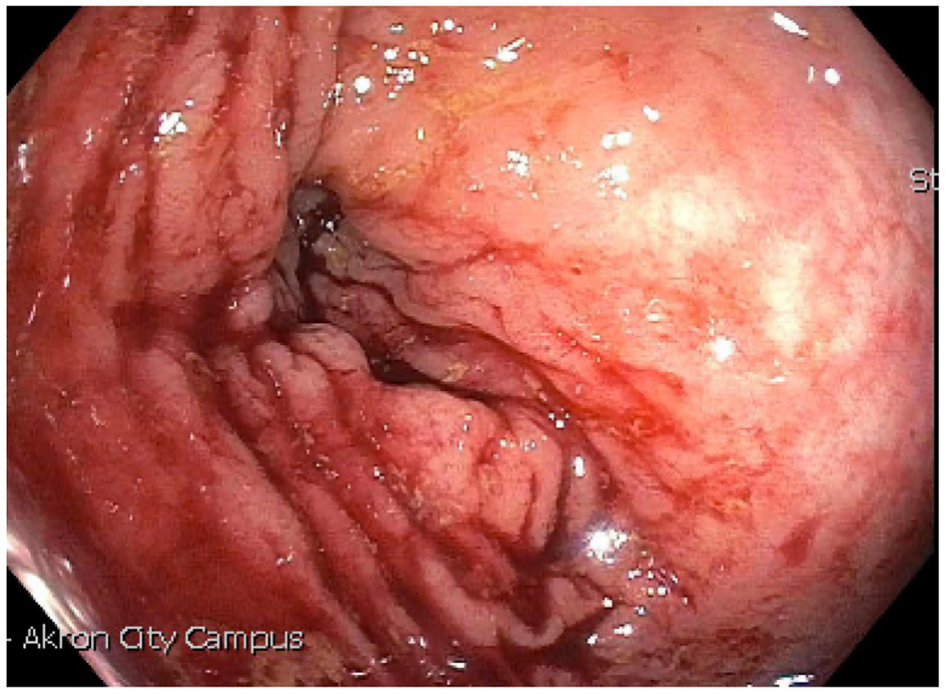
Endoscopic evidence of narrowing due to external compression in sigmoid colon.

**Figure 5:**
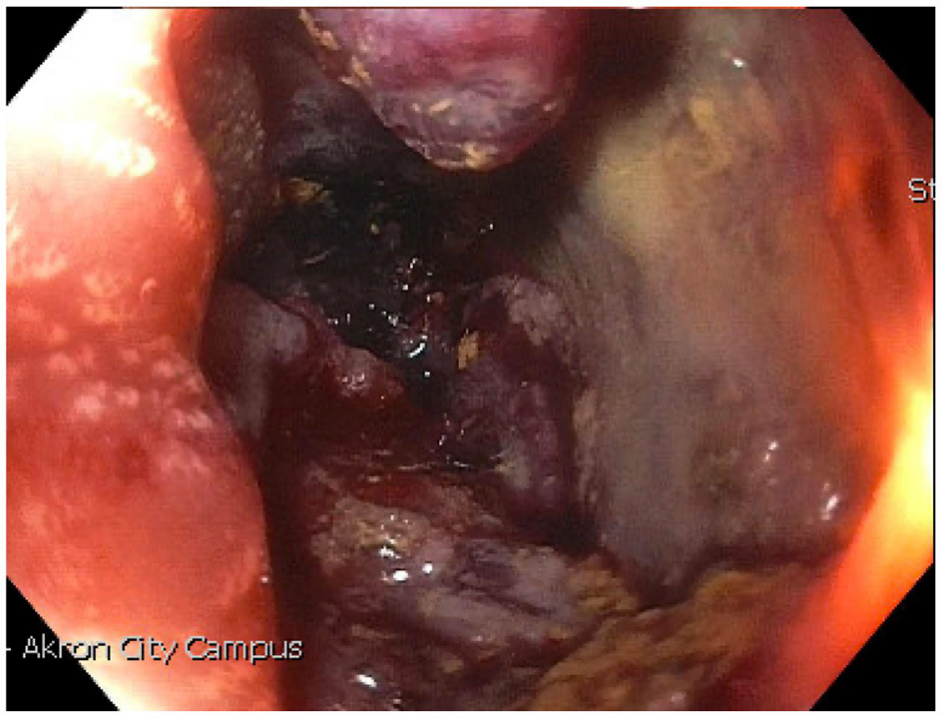
Mucosal ischemia at level of stenosis from external compression.

We present a case of primary peritoneal carcinosarcoma. This case originated from the mesenteric peritoneum of the sigmoid colon of a 48-year-old G8P8 woman of Amish descent. She has a history of a left modified radical mastectomy for stage IIB, pT2, pN1a, M0 triple negative breast cancer. She received adriamycin and cyclophosphamide ×4 along with weekly paclitaxel ×12. She presented complaining of bloating and increasing abdominal pain over the past 5 days. States complained of symptoms of bloating, fatigue, and abdominal distension for the past year which she thought was due to an abdominal hernia. She endorses a history of intermittent bloody bowel movements for approximately the past year. She was found to have a pelvic mass on physical exam. She underwent CT scan of the abdomen (Figures 1–3) and pelvis that demonstrated enlarged left periaortic lymph nodes, mild left hydronephrosis, narrowing of the sigmoid colon, and a large multi-cystic mass in the midline pelvis measuring 12.5 × 9.9 × 11.7 cm. She also underwent transabdominal US that demonstrated right ovary measuring 7.0 × 7.1 × 4.6 cm that is inseparable from a complex heterogeneous mass measuring 7.0 × 7.1 × 4.6 cm with a left ovary measuring 7.2 × 5.9 × 4.8 cm with an associated heterogenous mass measuring 6.4 × 6.0 × 5.4 cm. Due to the concern for sigmoid stenosis and history of intermittent bloody bowel movements she also underwent colonoscopy (Figures 4, 5) that showed stenosis due to external compression with mild mucosal ischemic changes without intraluminal lesion. Biopsies taken at that time did demonstrate invasive, moderately differentiated adenocarcinoma. She had elevated CEA (74.7 ng/ml, ref: 0–3 ng/ml) and CA 125 (53.7 U/ml, ref: 0–35 U/ml) with normal CA 19-9 (18 U/ml, ref: 0–37 U/ml). She underwent exploratory laparotomy that revealed a 15 cm mass to be originating from the sigmoid colon mesentery with normal, uninvolved ovaries, uterus and fallopian tubes. The mass was intimately involved, and appeared to invade the sigmoid colon, sigmoid mesentery, and the terminal ileum. Enlarged periaortic lymph nodes were appreciated and biopsied and several other small tumor deposits were noted in the mesentery. We performed en-bloc resection of the mass including a portion of the sigmoid colon, and ileum. A colorectal anastomosis was created, and a diverting loop ileostomy was brought up using the proximal and distal ends of the ileum to protect the colorectal anastomosis.

**Figure 6:**
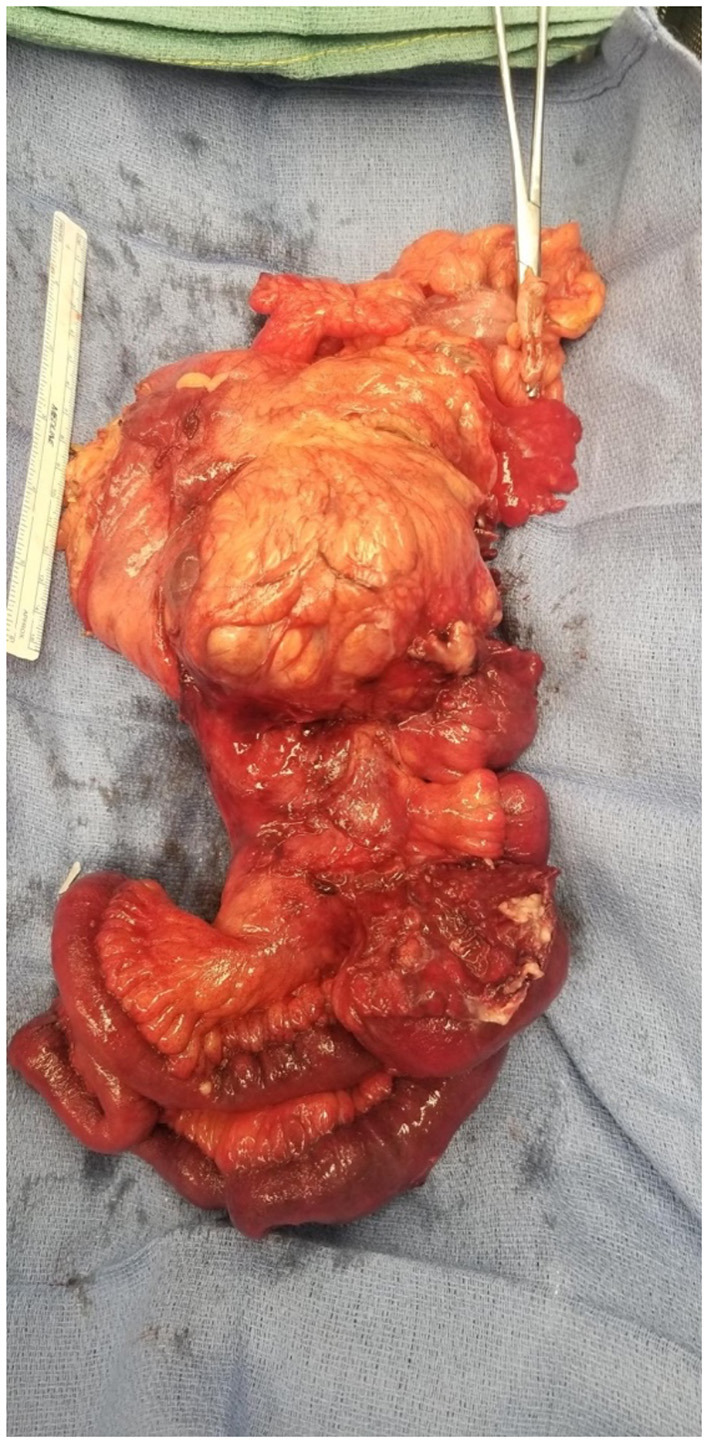
Gross pathology demonstrating mass with intimate association to sigmoid colon with involved mesentery and ileum resection. Posterior view.

**Figure 7:**
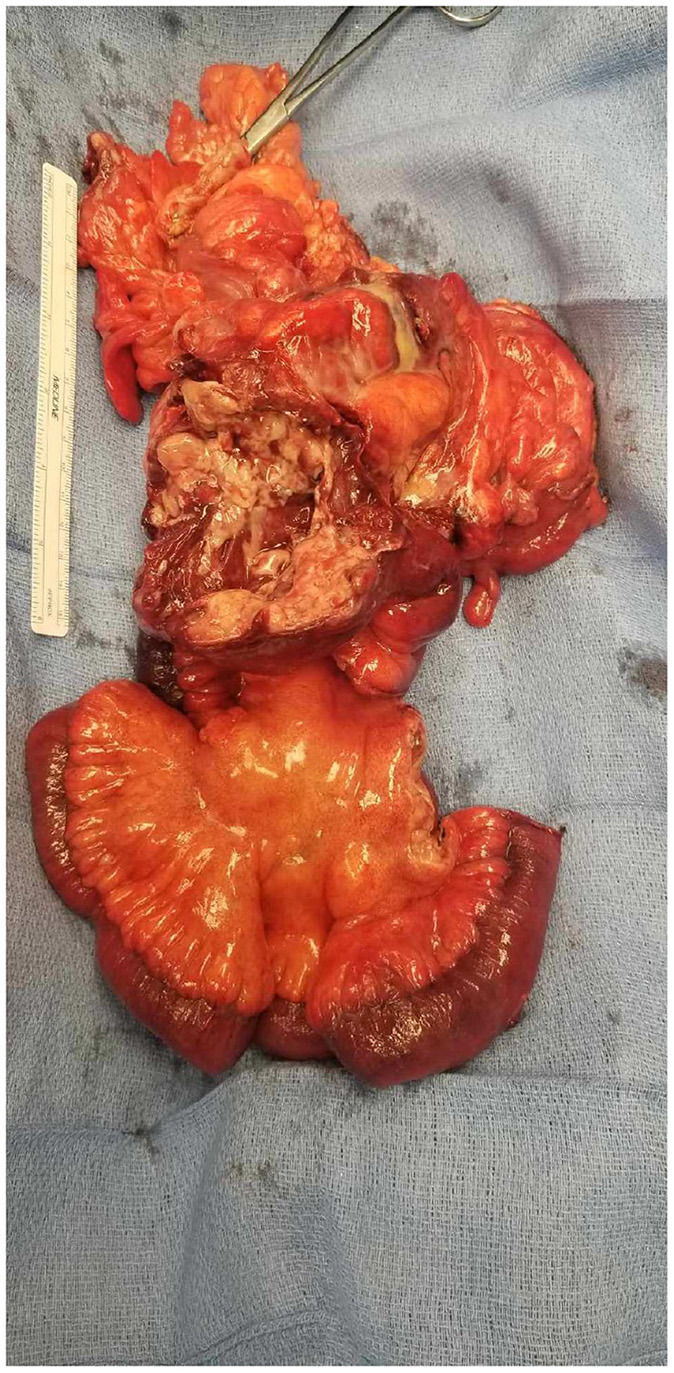
Gross pathology demonstrating mass with intimate association to sigmoid colon with involved mesentery and ileum resection. Anterior view.

**Figure 8:**
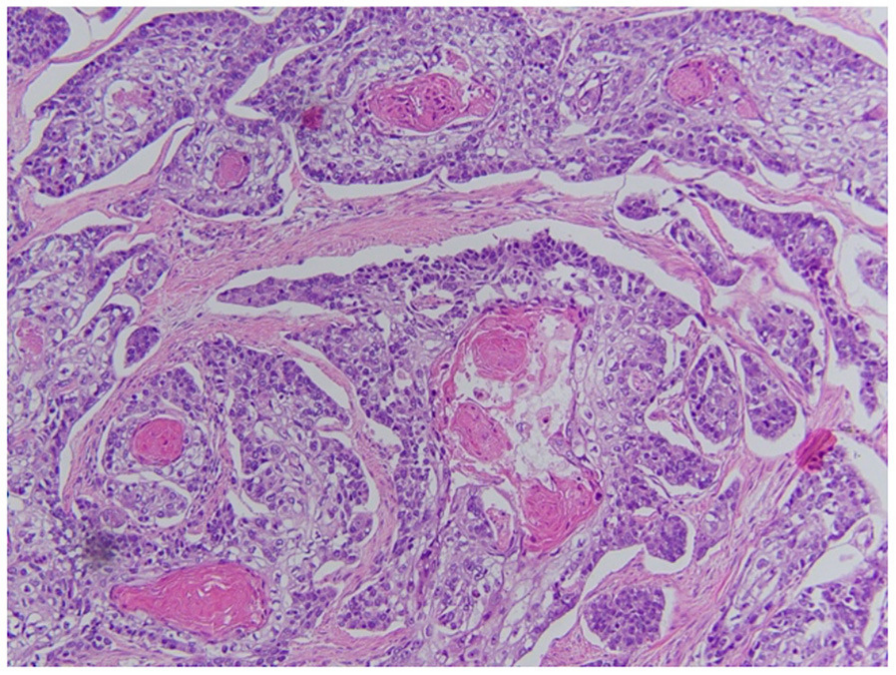
Squamous portion of the tumor. Demonstrates keratinization and intracellular bridges. There is a variation in size of the nuclei, there is nuclear atypia and prominent nucleoli.

**Figure 9:**
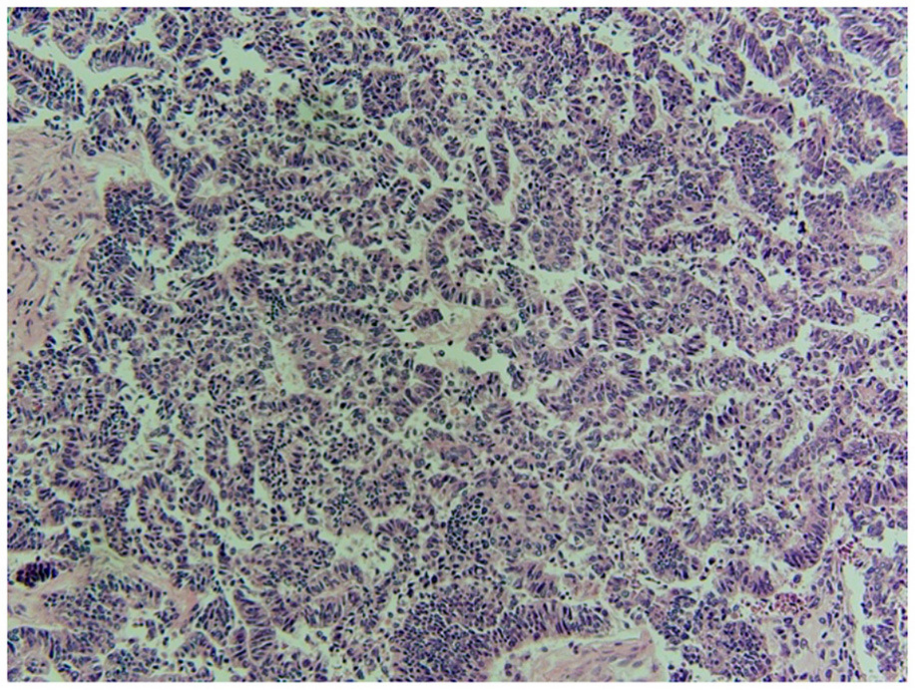
Chondroid portion of the tumor as well as cartilaginous matrix and various shapes/sizes of chondrocytes.

**Figure 10:**
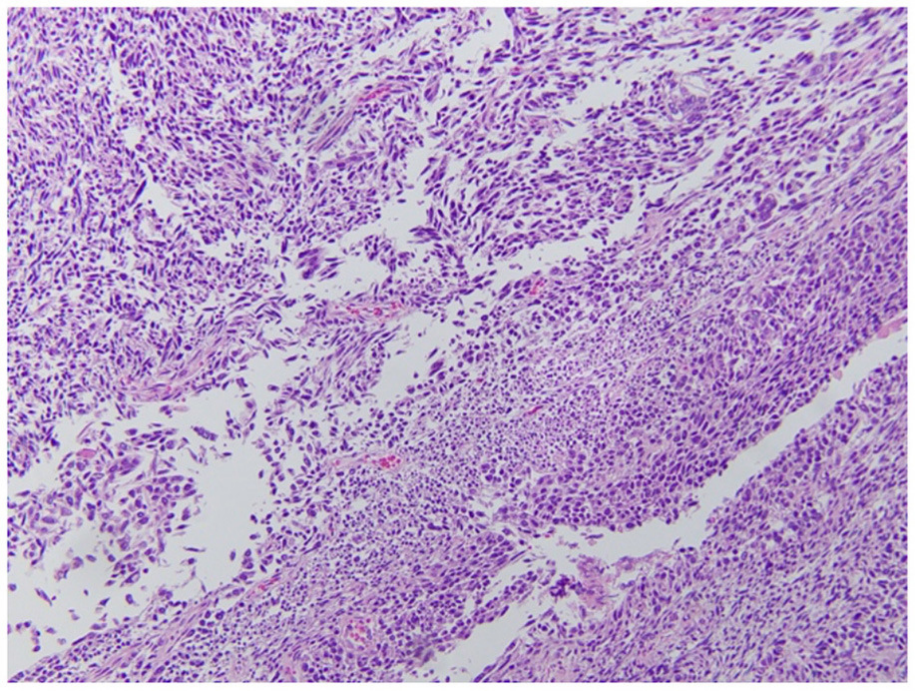
Spindle cell sarcoma component of the tumor.

**Figure 11:**
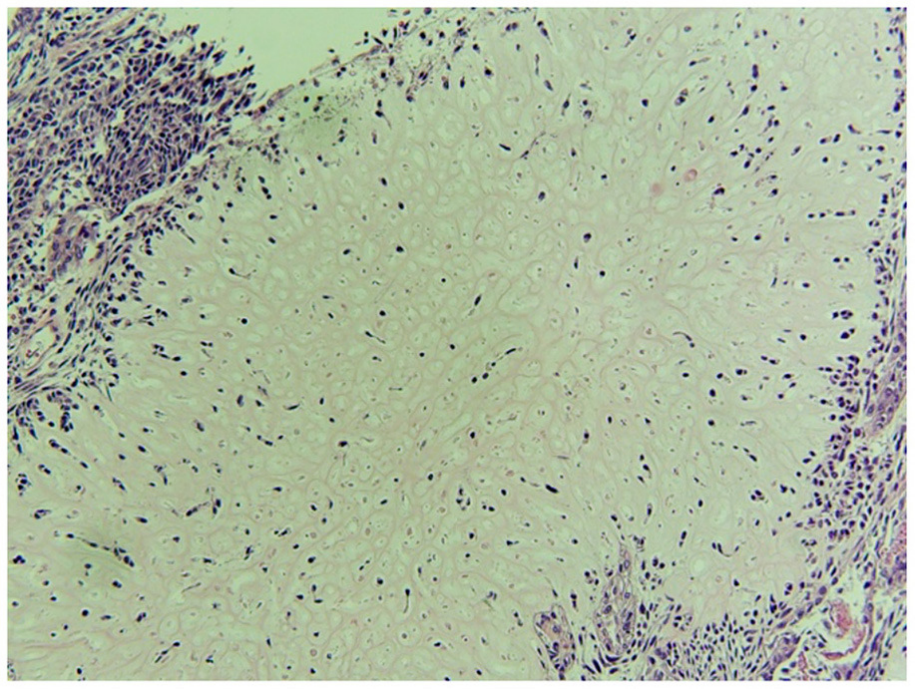
Adenocarcinoma features with gland formation by cuboidal to columnar cells. Nuclei with intracytoplasmic mucin droplets and some very large, atypical nuclei are seen.

Gross pathology (Figures 6, 7) revealed primary peritoneal carcinosarcoma measuring 7.5 × 6 × 4.5 cm. The tumor extensively involved the sigmoid mesentery and invaded the adjacent muscularis propria. Micro pathology (Figures 8–11) revealed much of the tumor consisting of malignant glands with a variable cuboidal to tall columnar lining and overtly malignant nuclear features. A minor keratinizing squamous carcinoma component was identified. Additionally, a malignant spindle cell stroma surrounds many of the glands creating a discrete biphasic appearance characteristic of carcinosarcoma. Heterologous chondrosarcoma is also identified. Immunohistochemical staining demonstrated pancytokeratin and p16 positive carcinomatous components; CK5/6, p63, p40 positive squamous differentiation; Cam5.2 and CEA positive in glandular differentiation; rare GATA-3 positive in sarcomatous component. The histologic features favor a Mullerian-type carcinosarcoma. Three out of 36 lymph nodes were positive for metastasis. The biopsied enlarged periaortic lymph node was negative for metastasis. Her post-operative course was not further complicated.

## Discussion/Conclusion

Peritoneal carcinosarcoma alone carries a poor prognosis with or without chemotherapy but that prognosis is even more grim with heterologous tumors vs. homologous as demonstrated by Kulkarni et al. with a mean survival of 4.8 vs. 9.1 months, respectively (5). There is not enough data currently to comment on improved survival with or without chemotherapy as it is nearly impossible to conduct a randomized trial on such a rare tumor. Leiser et al. demonstrated a complete response in 12 out of 30 patients with ovarian carcinosarcoma that underwent adjuvant chemotherapy with a taxane and platinum-based regimen (6).

Prior studies examining gene expression profiles in surgical samples of various primary human tumors have identified a potential link between triple negative breast cancer and MMMTs *via* the DNA repair enzyme Poly (ADP-ribose) polymerase-1 (PARP1) (7, 8). Unlike our patient, this link has been suggested to primary uterine and ovarian MMMTs rather than primary mesenteric tumors. Nonetheless, this suggests there may be a genetic component which contributes to the development of these malignancies, as the expression level of PARP1 is noted to be low in non-diseased human tissues (7). Further research might focus on elucidating a definitive genetic mutation, or syndrome, that can more easily identify patients predisposed to developing triple negative breast cancer and MMMTs. Such a discovery would allow for both earlier detection and intervention.

We present a multiparous 48-year-old female with a heterologous peritoneal carcinosarcoma who underwent surgical cytoreductive surgery. Her case is unique in the fact that she refused traditional recommended adjuvant chemotherapy for further treatment. She was seen by oncology who recommended adjuvant chemotherapy with taxane and platinum regimen. She preferred to trial natural remedies at home and will be followed with serial imaging with her first CT scan for surveillance being 3 months post-operative. This is a rare tumor with an often-grim prognosis despite adjuvant chemotherapy and is often found and diagnosed late in presentation.

## Data Availability Statement

The original contributions presented in the study are included in the article/supplementary material, further inquiries can be directed to the corresponding author/s.

## Ethics Statement

Written informed consent was obtained from the individual for the publication of any potentially identifiable images or data included in this article.

## Author Contributions

LE: paper compilation and initial surgical management. BS: paper compilation and research. BL: reference compilation and edits. TM: overseeing of project and primary surgeon. All authors contributed to the article and approved the submitted version.

## Conflict of Interest

The authors declare that the research was conducted in the absence of any commercial or financial relationships that could be construed as a potential conflict of interest.

## Publisher's Note

All claims expressed in this article are solely those of the authors and do not necessarily represent those of their affiliated organizations, or those of the publisher, the editors and the reviewers. Any product that may be evaluated in this article, or claim that may be made by its manufacturer, is not guaranteed or endorsed by the publisher.
